# The development and evaluation of the paediatric index of emotional distress (PI-ED)

**DOI:** 10.1007/s00127-015-1134-y

**Published:** 2015-12-19

**Authors:** Suzy O’Connor, Eamonn Ferguson, Terri Carney, Emma House, Rory C. O’Connor

**Affiliations:** Children and Young People’s Mental Health, Psychology Directorate, NHS Education for Scotland, 2 Central Quay, 89 Hydepark Street, Glasgow, G3 8BW UK; Mental Health and Wellbeing Research Group, Institute of Health and Wellbeing, College of Medical, Veterinary and Life Sciences, University of Glasgow Academic Centre, Gartnavel Royal Hospital, 1055 Great Western Road, Glasgow, G12 0XH UK; University of Nottingham, Nottingham, UK; NHS Ayrshire and Arran, Ayr, UK; Child and Adolescent Mental Health Services, Jersey, UK

**Keywords:** Sensitivity, Specificity, HADs, PI-ED, Emotional distress, Anxiety, Depression, Children/young people, Psychometrics

## Abstract

**Purpose:**

Current measures of anxiety and depression for children and young people (CYP) include somatic symptoms and can be lengthy. They can inflate scores in cases where there is also physical illness, contain potentially distressing symptoms for some settings and be impractical in clinical practice. The present study aimed to develop and evaluate a new questionnaire, the paediatric index of emotional distress (PI-ED), to screen for emotional distress in CYP, modelled on the hospital anxiety and depression scale.

**Methods:**

A school-based sample (*n* = 1026) was employed to examine the PI-ED’s psychometric properties and a clinical sample of CYP (*n* = 143) was used to establish its sensitivity and specificity.

**Results:**

Exploratory and confirmatory factor analyses identified a bi-factor model with a general emotional distress factor (‘cothymia’) and anxiety and depression as co-factors. The PI-ED demonstrated good psychometric properties and clinical utility with a cutoff score of 20.

**Conclusion:**

The PI-ED is a brief, valid and reliable clinical screening tool for emotional distress in CYP.

## Introduction

 Current measures of anxiety and depression for children and young people (CYP) include somatic symptoms (e.g. abdominal pain, tiredness, changes to appetite or dizziness) that could have an emotional or a physical basis. If symptoms have a physical basis but are scored as evidence of emotional distress, the scores may be artificially inflated. Conversely, emotional distress may be overlooked if somatic symptoms are viewed as physical in origin. For example, CYP who present in general practice with somatic signs of distress may be directed down a physical, rather than mental health care pathway. This could be avoided if a brief screening measure of emotional distress, that excluded somatic symptoms, was available. CYP with acute or chronic health conditions often present with associated emotional distress [1] and there are no measures available that can screen for this that do not include potentially confounding somatic symptoms [2]. For example, the Scottish Intercollegiate Guideline Network clinical guideline on the management of diabetes (guideline number 116) recommends that emotional distress is screened for routinely in this population, using the HADs for adults [3], but notes that there is no paediatric measure available that excludes somatic symptoms.

xA second issue with current measures of emotional distress in CYP is that they tend to be lengthy to complete, require either access to a computer or are dependent on the clinician consulting age-adjusted norms to determine clinical severity (e.g. the Strengths and Difficulties Questionnaire [4], the Achenbach Child Behaviour Checklist [5], the Beck Youth Inventories [6] the Children’s Depression Inventory [7], and the Spence Children’s Anxiety Scale [8]). Although existing measures have strengths, arguably the issues identified above may render their use impractical in busy clinical practice settings. While a shorter, more practical measure is desirable, it is important to demonstrate that it has clinical validity that is at least equal to existing measures before it is recommended for use.

To address the above issues, we developed and evaluated the PI-ED which is a brief, self-report screening tool for anxiety and depression that contains no somatic items and is suitable for CYP aged 8–16 years. The PI-ED is quick to complete, score and interpret, during a clinical session, using a cutoff score. The PI-ED was based on the HADs items because the HADs have good psychometric properties, its sensitivity and specificity compare favourably with the General Health Questionnaire, it correlates highly with other commonly used measures of anxiety and depression in adult samples and it is able to assess symptom severity and identify cases of anxiety and depression in somatic, psychiatric, primary care and general population samples [9]. Indeed, it is commonly used in adult populations to avoid the potential confound of physical symptoms and it has construct validity across different physical health conditions [10]. The present paper sets out to test the assertion that anxiety and depression in CYP can be identified, using the PI-ED, without reference to potentially confounding physical symptoms.

There is considerable debate about whether anxiety and depression can be distinguished reliably in CYP [11] given the high rates of symptom overlap, co-morbidity and similar treatment protocols for anxiety and depression. Indeed, these similarities had led to calls for Generalized Anxiety Disorder to be re-categorized as a Mood Disorder in DSM 5 [12]. Arguably anxiety and depression could be more usefully viewed as subordinate factors of a higher order construct, such as negative affectivity [13], internalizing disorders or cothymia [14, 15].

An alternative to this hierarchical approach to trait assessment [16] is suggested within the tripartite model of anxiety and depression [17, 18]. This model suggests that anxiety and depression are best conceptualized as consisting of both shared/general and unique factors [17]. In the Clark and Watson [18] model, these specific factors are Negative affect (NA), Positive affect (PA) and Physiological Hyper-arousability (PH). In this model, anxiety and depression are both linked to NA, with anxiety linked to high PH and depression to low PA (anhedonia), with comorbid anxiety and depression equating to high NA, low PA and high PH. Consistent with this model, it is possible that a general index of anxiety and depression is best conceived of consisting simultaneously of both a general factor (cothymia or NA) and two specific unique factors (i.e. anxiety and depression that may represent high PH and low PA). Such a factor structure would be specified as a bi-factor model [19]. A bi-factor model consists of a general factor on which all the symptoms load and a series of specific or unique factors on which specific target symptoms load. The general and specific factors are orthogonal. In terms of interpretation, the specific factors are considered to be residualized with respect to the general factor [19]. Such a model specification allows us to explore if there is predictive utility in the specific factors (anxiety and depression) once the general factor is taken into account [19]. If there is some utility in a specific factor, as well as the general factor, then whether or not the general or specific/unique factors are used for assessment and prediction would depend on the research/clinical question at hand.

Therefore, in the present study, we investigated whether emotional distress in this age group is best conceptualized as a single overarching emotional distress factor (e.g. cothymia [15], two distinct but correlated factors of depression and anxiety or as a bi-factor model, as suggested by the tripartite model.

### Aims

1. In phase one, we aimed to develop the PI-ED items, establish the psychometric properties of the PI-ED and examine the factor structure of emotional distress in CYP.
2. In phase two, we aimed to investigate the clinical validity of the PI-ED by establishing the sensitivity and specificity of the PI-ED against the computerized Diagnostic Interview Schedule for Children (C-DISC; Shaffer et al. [27]), the most widely used mental health interview, derive a clinically useful cutoff score and investigate the PI-ED’s test–retest reliability.

## Methods

### Phase one: development of PI-ED symptoms and their psychometric evaluation

#### Development of PI-ED items

The original HADS symptoms were re-worded in a child friendly manner, the introductory statements from the HADs were rewritten in a concrete and straightforward style and additional symptoms were devised to include key criteria outlined in the *DSM*-*IV*-*TR* [20], and the ICD 10th Revision [21]. Scoring was on a four-point scale, 3–0 (always, a lot of the time, sometimes, not at all). Three focus groups (18 participants recruited via snowballing from opportunistic general population samples), based broadly on Piagetian stages [22], were conducted with children and young people (CYP), aged 7–9 years, 10–12 years and 13–16 years to check that the measure was meaningful and easy to complete at each developmental stage. Symptoms were revised as a result of focus group feedback and the resulting measure was piloted on a consecutive sample of children and young people (*n* = 42 paediatric in/out patients aged between 9 and 16 years). Standard university and NHS ethical approvals were obtained.

This process resulted in 16 symptoms for the PI-ED (seven anxiety and nine depression), 12 of these were modified from the HADS (seven anxiety and five depression) and four were new, specially written depression symptoms (These items are shown in Table 1). The anxiety symptoms cover PH as suggested by the tripartiate model and the depression symptoms specifically cover anhedonia again as indicated by the tripartite model.

**Table 1:** The PI-ED symptoms

Symptom
a *Anxiety symptoms*—seven *symptoms*
I feel shaky or ‘wound up’ [A1]
I get a sort of frightened feeling as if something bad is about to happen [A2] (F) (Y)
I worry about things [A3] (F) (O)
I can chill-out and feel relaxed (r) [A4]
I get a sort of frightened feeling like ‘butterflies’ in my tummy [A5] (F)
I feel restless/fidgety as if I have to be on the move [A6] (Y)
I get panicky [A7] (F) (Y)
b *Depression symptoms*—nine *symptoms*
I still enjoy the things I used to enjoy (r)
I feel happy (r) [D1]
I feel sluggish/slowed down [D2] (O)
I look forward to fun things (r) [D3]
I cry/feel like crying [D4] (F)
I get annoyed easily [D5]
I feel good about myself (r) [D6] (F) (O)
I can enjoy a good book or computer game or TV programme (r)
I am lonely [D7] (Y)

The following 16 PI-ED symptoms were initially assessed as the basis for developing the PI-ED

(r) reverse scored

**Fig. 1:**
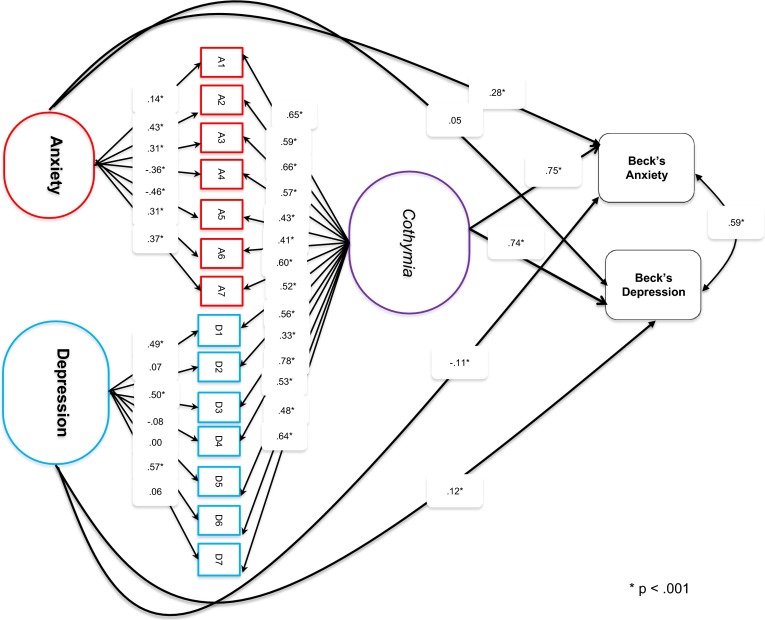
Structural equation model of the bi-factor PI-ED predicting beck’s childhood anxiety and depression

Code in the square brackets refers to the symptoms in Fig. 1

Cothymia is calculated by summing all the symptoms following reversal. As girls show differential symptom endorsement compared to boys (and younger to older) we suggest that clinicians also pay particular attention to these symptoms when making diagnoses and planning treatments

F, more likely to be endorsed by girls; Y, more likely to be endorsed by younger children; O, more likely to be endorsed by older children

#### Psychometric evaluation

*Participants* were recruited from schools in Scotland (West Central Scotland) and England (Nottingham). In West Central Scotland, 10 (38 %) out of 26 state secondary schools, one (14 %) out of seven independent schools and eight (45 %) out of 18 state primary schools took part in the study. In Nottingham, three (17 %) out of 18 state secondary schools, one (50 %) out of two independent schools and four (36 %) out of 11 primary schools participated. In the Scottish secondary schools, one class from first-fifth year was randomly selected while each primary school class was used. Within each class, eight pupils were randomly selected by the school (four male, four female). In Nottingham, classes were selected by the school and all pupils within that class were invited to take part. Universities of Stirling and Nottingham ethics committee approvals were obtained.

The initial sample comprised 1108 respondents; 47 % female (*n* = 521), 89 % White-UK and age range 7–17 years [mean 11.9 (2.33)]. In total, 21 respondents ticked the box to have their data destroyed, five were removed because they had not answered any of the 16 PI-ED symptoms and a further five were removed because they had omitted eight of the PI-ED symptoms. Of the remaining 1077 respondents, there was a small amount of missing data (0.3–0.6 %) on any single symptom. There were no associations between the pattern of missingness and age or sex. Listwise deletion resulted in a complete data set of 1026. The final sample comprised 51.9 % males with a mean age of 11.9 years (SD = 2.33 years) and was split randomly into two sub-samples of 513 participants. The first sample was used to conduct the exploratory factor analysis and initial factor development and the second for the confirmatory factor analysis.

#### Design

The design was cross sectional and employed a cluster sampling procedure (with school acting as the cluster variable).

#### Measures and procedure

In addition to the 16 symptom PI-ED, as part of the psychometric evaluation, participants also completed the Beck Youth Inventories for anxiety and depression–Second Edition (BYI-A&D [6]). These measures are commonly used in clinical practice, have good psychometric properties and they are suitable for use with CYP aged 7–18 years [6]. Administration took place during class time and was facilitated by a research assistant or by a classroom teacher. Data collection, for phase one, took place between 2008 and 2009. In the present study, internal consistency was high for BYI-A (α = 0.90) and BYI-D (α = 0.92).

### Statistical analyses

The sample was randomly split into two equal samples: one to conduct exploratory factor analyses and the other to conduct the confirmatory factor analysis. The psychometric analyses were conducted using M*Plus* 7 complex survey design routines to account for the clustering within school. The response scales are ordered-categorical and as such a weighted least-squares means and variance adjusted estimation (WLSMV) algorithm was used. A series of theoretical models was considered: (1) A single factor model to test if the PI-ED structure is a simple uni-dimensional model (general emotional distress factor), (2) a two-factor model, to test if the PI-ED structure consists of correlated anxiety and depression factors, and (3) a bi-factorial model, to test if the PI-ED consists of a general factor (cothymia) and specific factor(s). A hierarchical factor model of cothymia, with a general cothymia factor at the top of the factor hierarchy accounting for the covariance between anxiety and depression, was not considered because it would not be identified with only two lower order factors and would give results identical to a correlated two-factor model.

All reverse scored symptoms were re-coded prior to the analyses, so that in all cases high scores equated to greater distress. These models were examined using exploratory structural equation modelling (ESEM) procedures in the exploratory sample (a random 50 % of the psychometric sample) and confirmatory factor analytic (CFA) procedures in the confirmatory sample (the remaining random 50 % of the psychometric sample). Model fit was assessed using the comparative fit index (CFI), the Tucker–Lewis index (TLI), the root mean square error of approximation (RMSEA) and the Weighted Root Mean Square Residual (WRMR). Good fit is indicated with a CFI and TLI values close to 0.95, an RMSEA value close to 0.06 (and not significantly different from 0.05 indicating near fit) and a WRMR of one or less [23]. Regression models were conducted in Stata 13 with standard errors corrected for clustering within schools.

## Results (phase one)

Fit statistics for the competing factor models are shown in Table 2. In terms of the exploratory models, an initial one factor model was specified based on all 16 symptoms. This was not a good fit to these data. While all symptoms loaded significantly on the single factor, two symptoms had low loadings (‘I still enjoy things I used to enjoy’ and ‘I can enjoy a good book or computer game or TV programme’: loadings = 0.27 each). These symptoms were removed and the single factor model re-run, which yielded a slightly better fit, the remaining symptoms loading between 0.43 and 0.73 (all ps < 0.001). Interestingly, these symptoms reflected low PA or anhedonia from the tripartite model. The remaining 14 symptoms were retained for the exploratory factor analyses. The two-factor model was a good fit. However, there was no clear distinction between anxiety and depression, with 10 out of the 14 symptoms loading significantly on the first factor (loading for significance symptoms = 0.35–0.73, all ps < 0.001) and 10 symptoms loading significantly on factor 2 (loadings = −0.15 to 0.64, ps range = 0.018 to <0.001), with five of these symptoms loading significantly on both factors (ps 0.018 to <0.0001). Of these, three loaded primarily on factor 1 with loadings greater than 0.4 on factor 1 (0.50, 0.62, 0.71) and less than 0.4 of factor 2 (−0.15, 0.19, 0.27). The remaining two symptoms loaded less than 0.4 on both factors (factor 1 = 0.35, 0.36 and factor 2 = 0.28, 0.35). This suggests that anxiety and depression were not clearly differentiated. The bi-factor model was an excellent fit to these data and the loadings are shown in Table 3, where all symptoms load onto a general factor, but there is no clear differentiation between anxiety and depression.

**Table 2:** Fit statistics for the competing factor models

	Exploratory sample				Confirmatory sample (no methods factor)			Confirmatory sample (with methods factor)		
	1 factor (16 symptoms) (general factor)	1 factor (14 symptoms)	2 factor (correlated traits)	Bi-factor	1 factor	2 factor (correlated traits)	Bi-factor	1 factor	2 factor (correlated traits)	Bi-factor
CFI	0.90	0.92	0.98	0.99	0.87	0.88	0.95	0.96	0.96	0.98
TLI	0.88	0.91	0.97	0.98	0.85	0.85	0.92	0.95	0.95	0.97
RMSEA	0.07**	0.07**	0.04	0.04	0.078**	0.085**	0.06*	0.05	0.05**	0.04
WRMR	1.9	1.8	0.82	0.03^	1.8	1.8	1.1	1.01	1.05	0.77
*χ* ^2^ (*df*)	368.83 (104)	293.391 (77)	110.22 (64)	94.27 (52)	370.49 (77)	358.33 (76)	189.45 (63)	2263.20 (73)	2159.49 (72)	103.73 (59)
Association between latent factors			0.46**			0.87**			0.95**	

** *p* < .01, * *p* < .05 for difference of RMSEA from 0.05

^ The exploratory bi-factor model estimated using the BI-GEOMIN in M*Plus* seven provides an SRMR rather than a WRMR

**Table 3:** Symptom loadings for the bi-factor models

Symptoms	Exploratory sample			Confirmatory sample		
	General factor	Specific factor 1	Specific factor 2	General factor—cothymia	Specific factor 1 anxiety	Specific factor 2—depression
I feel shaky or ‘wound up’	0.63*	0.04	0.25*	0.66* (0.73*)	0.13* (−0.04)	
I get a sort of frightened feeling as if something bad is about to happen	0.60*	−0.21	0.22*	0.59* (0.63*)	0.48* (0.40*)	
I worry about things	0.71*	−0.31	−0.01	0.67* (0.68*)	0.31* (0.29*)	
I can chill-out and feel relaxed (r)	0.51*	0.40*	0.01	0.51* (0.36*)	−0.29* (−0.12*)	
I get a sort of frightened feeling like ‘butterflies’ in my tummy	0.53*	−0.41*	−0.01	0.42* (0.44*)	0.50* (0.51*)	
I feel restless/fidgety as if I have to be on the move	0.38*	−0.02	0.55*	0.43* (0.46*)	0.33* (0.30*)	
I get panicky	0.60*	−0.28*	0.17*	0.61* (0.63*)	0.33* (0.30*)	
I feel happy (r)	0.55*	0.41*	0.03	0.55* (0.47*)		0.54* (0.26*)
I feel sluggish/slowed down	0.56*	0.00	0.18*	0.52* (0.51*)		0.13* (0.51*)
I look forward to fun things (r)	0.42*	0.36*	−0.11	0.29* (0.21*)		0.58* (0.23*)
I cry/feel like crying	0.75*	−0.19	−0.09	0.77* (0.78*)		−0.14* (−0.19*)
I get annoyed easily	0.49*	0.15	0.34*	0.53* (0.51*)		−0.07* (0.14*)
I feel good about myself (r)	0.64*	0.42*	−0.08	0.42* (0.38*)		0.55* (0.08)
I am lonely	0.59*	0.06	0.05	0.62* (0.62*)		0.04 (0.13)

Figures in parentheses for the confirmatory bi-factor model are for the models with the methods factor included

* *p* < .05

The confirmatory models offer further support for the bi-factor structure of the PI-ED, with the bi-factor model having the best fit to these data (Table 2). While the bi-factor model is not strictly nested within the two-factor model, it is within a hierarchical factor model. As detailed above the hierarchical factor model is not identifiable unless one of the higher order loadings is initially fixed to unity or equality constraints are used. Doing this provides the same fit statistically as the correlated two-factor model. We thus specified such a hierarchical model and used it to examine if the fit for the bi-factor model is an improvement over a two-factor representation. The Chi square difference was significant (Δ*χ*^2^ = 195.251 (13) *p* < .0001) indicating that the bi-factor model is the better fit. The factor loadings for the CFA bi-factor model (Table 3) again show a clear general factor with two weaker specific factors of anxiety and depression.

Given that there are four negatively worded symptoms on the specific factors an additional methods factor, orthogonal to the other factors, constituting these negatively worded symptoms was added to the confirmatory models. The results are also presented in the last three columns of Table 2 (Confirmatory Sample (with methods factor)). We calculated the Chi square difference using the same procedure as above and the difference was significant (Δ*χ*^2^ = 74.62 (13) *p* < .0001). Again the bi-factor model was the best fit and the loadings based on the model with the methods factor are given in Table 3 in parentheses.

Taken as a whole, these analyses support a bi-factor model with a general cothymia (distress) construct and two weaker specific factors of anxiety and depression. On the specific factors, depression is the weakest with two non-significant loading symptoms. For the whole sample, cothymia had an internal reliability of 0.83, and anxiety and depression had reliabilities of 0.74 and 0.70, respectively.

## Initial validation and fairness

### Construct validity

Ordinal Least-Squares (OLS) regression models (with standard errors corrected for clustering within school) were conducted with cothymia, as well as the specific sub-factors of anxiety and depression, as outcomes with both BYI-D and BYI-A entered as predictors using the whole sample (see Table 4). This model indicated that cothymia was associated with both BYI-D (*Β* = 0.26) and BYI-A (*Β* = 0.28), and the size of these associations was not significantly different from each other (*F*_(1, 26)_ = 0.08, *p* = .76). There was also evidence for double dissociation for the specific sub-factors of anxiety and depression. Anxiety showed a significantly higher association with BYI-A (*Β* = 0.24) than BYI-D (*Β* = 0.05; *F*_(1, 26)_ = 25.9, *p* < .0001), with the converse true for depression, with depression showing a higher association with BYI-D (*Β* = 0.21) than BYI-A (*Β* = 0.04; *F*_(1, 26)_ = 29.5, *p* < .0001).

**Table 4:** Initial validity analyses summarizing the associations between the BYI-A, BYI-D and PI-ED

	PI-ED cothymia		PI-ED anxiety		PI-ED depression	
	*B* (95 % CI)	*β*	*B* (95 % CI)	*β*	*B* (95 % CI)	*β*
BYI-A	0.28*** (0.21, 0.35)	0.41***	0.24*** (0.20, 0.28)	0.61***	0.04* (0.006, 0.08)	0.12*
BYI-D	0.26*** (0.20, 0.33)	0.39***	0.05** (0.01, 0.09)	0.13**	0.21*** (0.18, 0.25)	0.60***
*R* ^*2*^	0.58***		0.52***		0.49***	

Standardized beta coefficients *β* were estimated using Long and Freeses’ *listcoef* command in Stata 13. The *n* for these models was 1015 due to missing data in the BYI-D and BYI-A

* *p* < .05, ** *p* < .01, *** *p* < .001

However, since the specific factors are residualized relative to the general factor, it is necessary to show if the specific PI-ED factors (anxiety and depression) still predict BYI-D and BYI-A, with a double dissociation, in the presence of cothymia (the general factor). Thus, following DeNars’ (2013) recommendations, a structural equation model was specified (see Fig. 1) with the latent factors of the bi-factor model specified to predict scores on Beck’s Youth Inventories (BYI) for anxiety and depression. This model had a good fit to these data (CFI = 0.96, TLI = 0.95, RMSEA = 0.056 with a *p* = .053). Examining the paths from the latent factors to Beck’s anxiety and depression youth inventories scores indicates clearly that cothymia is a strong predictor of both. However, both specific factors have some utility with PI-ED anxiety predicting BYI anxiety but not BYI depression. The dissociation for PI-ED depression is less clear. This predicts BYI depression but also anxiety where the association is negative. As such the depression sub-scale of the PI-ED should be treated with some caution when used clinically. However, the cothymia general factor is strong and should be used. The PI-ED anxiety factor may also have some clinical utility.

### Equivalence (MIMIC model)

We explored equivalence with respect to sex and age. We use Multiple-Indicator Multiple-Causal (MIMIC) Models to achieve this. MIMIC models are a type of CFA model where the latent factors and symptoms are simultaneously regressed on demographics (and other relevant covariates depending on the research question). Significant effects on the latent factors indicate how the mean level of a factor varies as a function of demographics and significant direct effects on symptoms represent a differential item functioning (DIF) indicating how the symptom responses vary as a function of demographics [24, 25]. Significant DIF indicates if there is measurement invariance in terms of specific symptom endorsement, with respect to demographic characteristics. Following recommended procedures, a standard CFA model was specified first to represent the main features of the measurement model (*base model*) and the MIMIC model was specified on the base model [24, 25]. As there is evidence that depression, anxiety and cothymia vary as a function of age and sex, the MIMIC model was specified with age and sex as covariates of anxiety, depression and cothymia latent factors. To identify additional DIF effects, each symptom used in the study was regressed on age and sex. Significant effects were additionally entered into the MIMIC model. Using a backwards elimination procedure, non-significant effects were removed from the MIMIC models until a final best fitting model was observed [24–26]. All models were estimated using WLSMV estimation in M*plus* 7 and were based on the full sample.

The final best fitting MIMIC model was a good fit to the data (CFI = 0.96; TLI = 0.95; RMSEA = 0.047 [*p* RMSEA ≤ 0.05 is 0.79]). All symptoms loaded significantly on cothymia. The seven anxiety symptoms loaded significantly on the anxiety factor and four of the seven depression symptoms loaded significantly on the depression factor (essentially resembling the results of Fig. 1). In terms of age and sex, the MIMIC model indicated that boys scored higher on depression than girls (*β* = *0*.21, *p* < .001). A number of significant DIF were observed for sex, indicating that girls are more likely to endorse the following symptoms: (1) “I get a sort of frightened feeling as if something bad is about to happen” (*β* = −0.21, *p* < .001), (2) “I worry about things” (*β* = −0.23, *p* < .001), (3) “I get a sort of frightened feeling like ‘butterflies’ in my tummy” (*β* = −0.36, *p* < .001), (4) “I get panicky” (*β* = −0.20, *p* < .001), (5) “I cry/feel like crying” (*β* = −0.31, *p* < .001) and (6) “I feel good about myself” (but reversed to indicate not feeling good) (*β* = −0.20, *p* < .001). The first four symptoms are from the anxiety and the latter two from the depression scale. Thus, there is differential endorsement of anxiety symptoms by sex with girls more likely to endorse fear and panic symptoms than boys and a differential endorsement of depression symptoms by sex with girls more likely to endorse crying and low self-worth symptoms than boys. There was also a significant DIF for age. Younger children are more likely to endorse the following symptoms: (1) “I get a sort of frightened feeling as if something bad is about to happen” (*β* = −0.08, *p* = .05), (2) “I feel restless/fidgety as if I have to be on the move” (*β* = −0.09, *p* < .001), (3) “I get panicky” (*β* = −0.10, *p* < .001), and (4) “I am lonely” (*β* = −0.11, *p* < .001). Older children are more likely to endorse the following symptoms: (1) “I worry about things” (*β* = 0.08, *p* = .026), (2) “I feel sluggish/slowed down” (*β* = 0.07, *p* = .021), and (3) “I feel good about myself” (but reversed to indicate not feeling good) (*β* = 0.12, *p* = .003). Thus, the DIF clearly shows that the effects of age on how individual symptoms are endorsed varies, with younger children endorsing symptoms about fear and panic and older children rating symptoms on worry, image and motivation. It seems that age and sex affect the likelihood of different symptom endorsements. One way to interpret this is in terms of heterogeneity of cothymia. That is, girls are more likely than boys to present with a type of cothymia that is characterized by fear, panic, crying and self-worth. Younger children are more likely also to have cothymia that focuses on fear and panic and less so on worry and self-worth.

The finding that boys score higher on the latent depression factor is at odds with much of the literature. This likely reflects the simultaneous assessment of effects of sex on symptoms and latent factors. Indeed, when the effect of sex on depression is removed from the MIMIC model, the model still provides a good fit to the data (CFI = 0.96; TLI = 0.94; RMSEA = 0.047 [*p* RMSE ≤ 0.05 is 0.75]) and the DIF results remained robust.

This suggests that the PI-ED is used primarily to assess cothymia and scored as in Table 1. As specific symptoms are more likely to be endorsed by girls (see Table 1), clinicians should interpret girls’ cothymia in this context and expect them to score higher than boys on these specific symptoms. Similarly, younger children should be expected to score higher on the symptoms indicated in Table 1. So, although the overall score is simple, clinicians should examine these specific symptoms in detail when diagnosing boys and girls, and younger and older children.

## Phase two: clinical validity

A clinical sample was recruited from one Scottish Health Board area to assess the PI-ED’s diagnostic sensitivity and specificity, to derive a clinically useful cutoff score and investigate the PI-ED’s test–retest reliability. A consecutive sample of young people from eight hospital paediatric outpatient departments was obtained (*n* = 113). In addition, a clinician-targeted sample of CYP who presented with low mood/anxiety from a Child and Adolescent Mental Health Service (CAMHS) (*n* = 5) and a Hospital-based Paediatric Psychology service (*n* = 25) was recruited. Standard NHS research ethical approval was obtained.

In total, 143 CYP [mean age 12.2(2.5) years, age range 8–17 years; 52 % male] were recruited. The demographics of this sample compare favourably with the psychometric sample (phase one) who had a mean age of 11.9 years (SD = 2.3) and were 51.9 % male. 97 lived with both of their parents, 32 with one parent, nine with one parent plus a step-parent, two with other family members and three ‘other’. 140 were White-UK, two White-Other and one reported Mixed Ethnicity. We did not have ethical approval to compare the characteristics of these respondents with non-participants.

### Design

This was a cross-sectional study with a 1-week follow-up of a subsample.

### Measures

The 16 symptom PI-ED, the BYI-A&D and the reference standard measure, the computerized Diagnostic Interview Schedule for Children (C-DISC; [27, 28], were employed. The C-DISC was employed as the reference standard because it is the most widely used mental health interview for use in clinical and non-clinical populations in this age group [27]. Although participants completed the 16 symptom PI-ED, informed by the factor analyses of Phase one, all analyses were conducted on the 14 symptom PI-ED.

### Procedure

At time one all participants completed the PI-ED, BYI-A&D (counter-balanced) and then the C-DISC was presented on a laptop computer. All measures were administered on NHS premises. 1 week later, at time two, the first 100 participants re-completed the PI-ED within their own homes. It has been recommended, as rule of thumb, that a minimum sample size for test–retest in this type of context is 50 [29]. To err on the side of caution, we doubled that. The remaining 43 participants were not invited to take part at time two as 100 participants were deemed sufficient to investigate test–retest reliability. Data collection for the clinical validation phase was completed between 2010 and early 2012.

## Results (phase two)

Sensitivity and specificity for different cutoffs for the general PI-ED factor (cothymia) were examined. The optimal cutoff was defined as a score that maximized the Youden Index [30–32]. All analyses were conducted using Stata 13 and SPSS 20.

The PI-ED cothymia index had a mean of 12.3 (SD = 7.12; α = 0.88), the PI-ED depression index had a mean of 6.2 (SD = 3.9, α = 0.81) and the PI-ED anxiety index had a mean of 6.2 (SD = 3.8, α = 0.79). As the structure of the PI-ED is bi-factor (a general factor and two specific sub-factors that represent anxiety and depression), the sensitivity and specificity analyses focused on those with a definite diagnosis of either generalized anxiety disorder (GAD), major depressive disorder (MDD) or both (based on the C-DISC diagnoses). Within the sample, there were other diagnoses, however, we focused on anxiety and depression given the PI-ED, like the HADs, was not designed to identify these more specific types of anxiety. Seven participants had their diagnosis missing on either GAD or MDD. Only four participants achieved a diagnosis of cothymia (a definitive diagnosis of both GAD and MDD). This number was too small to conduct the sensitivity and specificity analyses so we examined all participants who had a definitive diagnosis of either GAD (*n* = 4) or MDD (*n* = 11)^1^ or were comorbid for both (*n* = 4). Thus, there were 19 cases who scored one if participants had GAD, MDD or both while those who did not have a diagnosis of GAD, MDD or both were scored 0 and made up the comparison group (*n* = 116).

Psychopathology was observed in the comparison group. Table 5 compares the cases and comparison groups on the prevalence of the other disorders defined by a definitive diagnosis. Importantly, the relative numbers of other diagnoses were small and they tended not to vary systematically across the groups. However, cases were significantly older and there was a higher percentage of females. The maximum Youden index (0.77) was for a score of 20 or greater on the PI-ED which corresponds to a sensitivity of 0.83, a specificity of 0.93 and a correct classification rate of 92 %. For depression, the maximum Youden score (0.75) indicated a cutoff of 8 or greater (sensitivity = 94.44 %, specificity = 80.76 %, correct classification = 82.58 %). For anxiety, the maximum Youden score (0.74) indicated a cutoff of 9 or greater (sensitivity = 89.44 %, specificity = 85.22 %, correct classification = 85.82 %).

**Table 5:** Comparison of the cases with respect to diagnosis, age and sex

	Diagnosed/*n*	Comparison Diagnoses/*n*	Cases Diagnoses/*n*	
Social phobia	3/89	0/81 (37 %)	3/8 (0 %)	
Separation anxiety	9/122	3/109 (2.8 %)	6/13 (46.2 %)	*Z* = 5.6, *p* = .07
Specific phobia	27/111	19/97 (19.6 %)	8/14 (57.1 %)	*Z* = 3.2, *p* = .24
Panic disorder	6/122	2/108 (1.9 %)	4/14 (28.6 %)	*Z* = 4.3, *p* = .049
Agoraphobia	14/123	9/109 (8.3 %)	5/14 (35.7 %)	*Z* = 3.0, *p* = .11
Selective mutism	1/132	1/116 (0.9 %)	0/16 (0 %)	
OCD	17/132	5/114 (4.4 %)	12/18 (66.7 %)	*Z* = 7.0, *p* = .13
PTSD	3/129	1/111(0.9 %)	2/18 (11.1 %)	
Dysthymia	0/134	0/116 (0 %)	0/18 (0 %)	
Mania	0/126	0/113 (0 %)	0/13 (0 %)	
Hypomania	2/128	1/114 (0.9 %)	1/14(7.1 %)	
Age		11.7 (2.5)	14.2 (1.7)	*t* (133) = 4.0, *p* < .001
Sex		47 % female	74 % female	*χ* ^2^ (1) = 4.5, *p* = .035

The Ns vary as the analysis focused on those with either a definitive diagnosis or no diagnosis; the C-DISC intermediate diagnosis was not included

As the comparison group contains some diagnosed patients, we also ran the Receiver Operating Characteristic (ROC) curve analysis when these were removed from the comparison group. The results are the same as those reported above. This suggests that a cutoff score of 20 or greater will identify those who are at risk of developing comorbid anxiety and depression, 8 or greater for depression and 9 or greater for anxiety. As noted above, caution should be employed with the PI-ED depression subscale as a screening tool.

A sub-sample of 100 participants completed the PI-ED at times one and two. The PI-ED at time two demonstrated acceptable internal reliability (α = 0.86) and the Spearman’s rho test–retest correlation was 0.81 (*p* < .0001). For PI-ED depression, it was 0.77 (*p* < .001) and for anxiety it was (0.71, *p* < .001). Anxiety and depression each showed acceptable internal reliability at time 2 with alphas of 0.79 and 0.74, respectively.

## Discussion

In this two phase study, we aimed to (1) develop the PI-ED items, establish the psychometric properties of the PI-ED and examine the factor structure of emotional distress in CYP and, (2) establish the sensitivity and specificity of the PI-ED by comparing it with the gold standard C-DISC (Shaffer et al. [27]) and derive a clinically useful cutoff score and investigate the PI-ED’s test–retest reliability.

Overall, our results suggest that the PI-ED is a valid and reliable measure of emotional distress in CYP. The results also contribute to the debate about how best to conceptualize emotional distress in CYP; as separate factors or as a single factor such as ‘cothymia’ [15] or as a bi-factor model. High comorbidity rates, coupled with similar treatment protocols for anxiety and depression, have led to a debate questioning the utility of the separate classifications of anxiety and depression and a call for the adoption of a unifying construct such as ‘cothymia’ [15]. The present findings are consistent with such calls, and add to that debate by indicating that a bi-factor model best represents the structure of anxiety and depression in this age group. This suggests that emotional distress in this age group consists of a general cothymia factor and although there is some evidence for separate anxiety and depression factors, there is little residual variance explained by the depression sub-factor. Thus, we suggest that cothymia is assessed primarily by the PI-ED and this is a simple summed score as detailed.

The MIMIC model showed that girls were more likely to endorse specific symptoms linked to fear, panic, crying and low self-worth than boys. This indicates that girls report a specific sub-type of cothymia distinct from boys. This corresponds with the literature in adults which finds that women are more likely to report emotional distress than men [33] and suggests that CYP are similar to adults in this regard [34]. In the clinical validation phase, a cutoff score of 20 was established as a useful cutoff score that allows the PI-ED to be used as a brief screening tool for CYP. Thus, we suggest that clinicians use the score of 20 as an initial screen and then explore these specific symptoms that show differential responding across girls and boys and use these to guide specific interventions. While this study shows that girls endorse specific cothymia symptoms differently to boys, it does not say why this is (see [35] for discussion). As Gallo et al. [35] point out these differences could reflect psychological/socio-cultural (e.g. sex roles or willingness to disclose) or biological (e.g. hormonal changes at puberty) factors and future work needs to explore these. The advantage of the MIMIC model is that it helps to identify the specific symptoms that are of interest rather than just the global diagnosis of cothymia. As such, the search for causal mechanisms to explain the differential symptom endorsement can be more focused. The same argument applies to the variability observed with age.

While all symptoms loaded significantly on the single factor, two symptoms had low loadings (‘I still enjoy things I used to enjoy’ and ‘I can enjoy a good book or computer game or TV programme’: loadings = 0.27 each). These symptoms were removed and the single factor model re-run, which yielded a slightly better fit. Arguably, these symptoms tap a key element of depression, namely anhedonia. This is also linked to low PA and is a key component of the tripartite model of anxiety and depression [18]. This leads to the question, why did they not hold up psychometrically? It may be that these symptoms were worded in such a way that they were not understandable for CYP. CYP grow out of activities as they develop so the question about still enjoying the things one used to enjoy may not make sense to them. It is also possible that the symptom about enjoying a good book and computer game is somewhat dated for today’s CYP. In any case, the symptom, ‘I look forward to fun things’ was retained and itself taps the concept of anhedonia. Alternatively, as depression develops later in adolescence after anxiety [36], it may be that many aspects of depression are not ‘familiar’ to this age group. This may account for why the anhedonia symptoms dropped out but also explain the slightly weaker psychometric properties for the depression sub-scale in this age group. Indeed, Anderson and Hope [37] raise some concerns about the tripartite model as applied to younger populations.

### Limitations

First, the samples lacked ethnic diversity. Further assessment of the PI-ED across diverse ethnic categories would, therefore, be a useful next step. Second, the present study only used the MDD and GAD diagnoses derived from the C-DISC so it might be useful to include all possible diagnostic categories under the terms ‘anxiety’ and ‘depression’ within DSM-5 and ICD-10 in future research. Third, although the present study highlighted the utility of the PI-ED, in future research, it would be interesting to compare the PI-ED with existing brief screening tools which assessed physical symptoms to determine whether the PI-ED demonstrates better sensitivity and specificity.

## Conclusion

Overall, these results suggest that the PI-ED is a valid, reliable measure of emotional distress in CYP that is brief to complete and score; it has a clear cutoff score of 20 and does not contain somatic symptoms. Our findings add to the debate about how best to conceptualize emotional distress in CYP and suggest that a bi-factorial structure termed ‘cothymia’ best captured these data.

## Footnotes

1 For 7 of these 15 patients there was only data available on either MDD (*N* = 4) or GAD (*N* = 3), the data on the other diagnosis category was missing. For the remaining 8 of these 15 participants data were available on both MDD and GAD.
